# The Pulse Beneath the Mass: Detection of a Bronchial Artery Pseudoaneurysm Using Endobronchial Ultrasound With Color Doppler

**DOI:** 10.1016/j.chpulm.2026.100261

**Published:** 2026-04-29

**Authors:** Haley N. Ferguson, Cory M. Powers, W. Graham Carlos, Joseph P. Smith, Edwin J. Jackson, Brent A. Bagley

**Affiliations:** a Internal Medicine Residency Program, Indiana University School of Medicine, Indianapolis, IN; b Division of Pulmonary and Critical Care Medicine, Indiana University School of Medicine, Indianapolis, IN

A 36-year-old man with a history of methamphetamine use and vaping presented with a 1-week history of progressive hemoptysis, fever, chills, weight loss, and pleuritic chest pain. His symptoms began with a cough-producing blood-streaked sputum, which progressed to large-volume hemoptysis (> 300 mL). He denied any palpable lymphadenopathy, night sweats, dyspnea, orthopnea, leg swelling, gastrointestinal complaints, dysuria, hematuria, or recent sick contacts. Physical examination was notable for poor dentition, tachycardia, and rhonchi. Although initially hemodynamically stable on room air, his hemoptysis worsened despite treatment with nebulized tranexamic acid. This prompted intubation for airway protection and transfer to the ICU.

CBC count revealed leukocytosis (26,600 cells/cm^3^) with neutrophilic predominance. Other laboratory tests, including coagulation profile, electrolytes, and renal and liver function panels, were within normal limits. Autoimmune workup, including testing for vasculitis and connective tissue disease, was negative. Chest radiography demonstrated new right perihilar infiltrates not seen on imaging 2 weeks prior. CT angiography (CTA) of the chest revealed significant mediastinal adenopathy with encasement of the right-sided pulmonary arteries and the right middle and lower lobe bronchi, along with obstruction of the right middle lobe bronchus (Fig 1). The patient was started on broad-spectrum antibiotics for empirical treatment of pneumonia.

**Figure 1:**
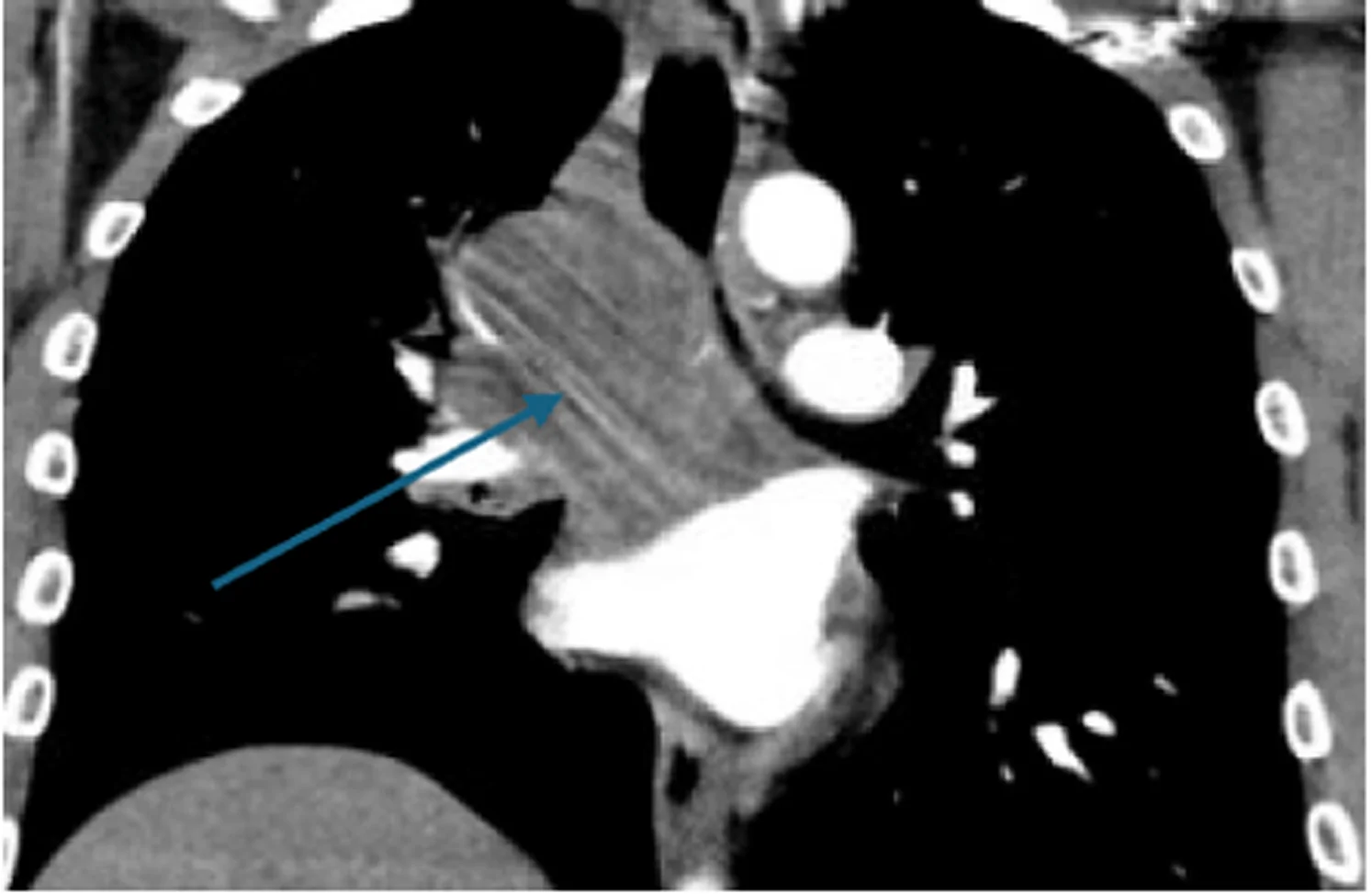
Coronal section of the CT angiography chest showing mediastinal adenopathy (blue arrow) with encasement of right-sided pulmonary arteries and right middle and lower lobe bronchi with obstruction of the right middle lobe bronchus.

*Question 1: What is the differential diagnosis for a patient presenting with progressive hemoptysis, fever, weight loss, and mediastinal lymphadenopathy?*

*Question 2: Can* *endobronchial ultrasound (EBUS)* *with color Doppler be used to identify pseudoaneurysms in patients with hemoptysis undergoing bronchoscopy?*

*Answer:* Bacterial mediastinitis, fungal mediastinitis such as histoplasmosis or blastomycosis, TB, sarcoidosis, vasculitis, lymphoma, or other malignancy.

Bronchoscopy revealed purulent secretions and clotted blood in the right middle and lower lobes; however, no active bleeding was observed. A protruding endobronchial lesion was noted on the anterior surface of the right mainstem bronchus. Infectious workup on bronchoalveolar lavage samples, including testing for acid-fast bacilli, endemic fungi, and bacterial pathogens, was later determined to be negative.

Given the presence of surrounding mediastinal lymphadenopathy, EBUS was performed. EBUS identified lymphadenopathy in the right hilum (10R region), which was biopsied. Rapid on-site evaluation revealed necrosis and bacteria, without evidence of granulomatous inflammation or malignancy. Additional fine needle aspirate cultures were sent from this site. An adjacent vascular structure was also noted before biopsy and avoided (Fig 2).

**Figure 2:**
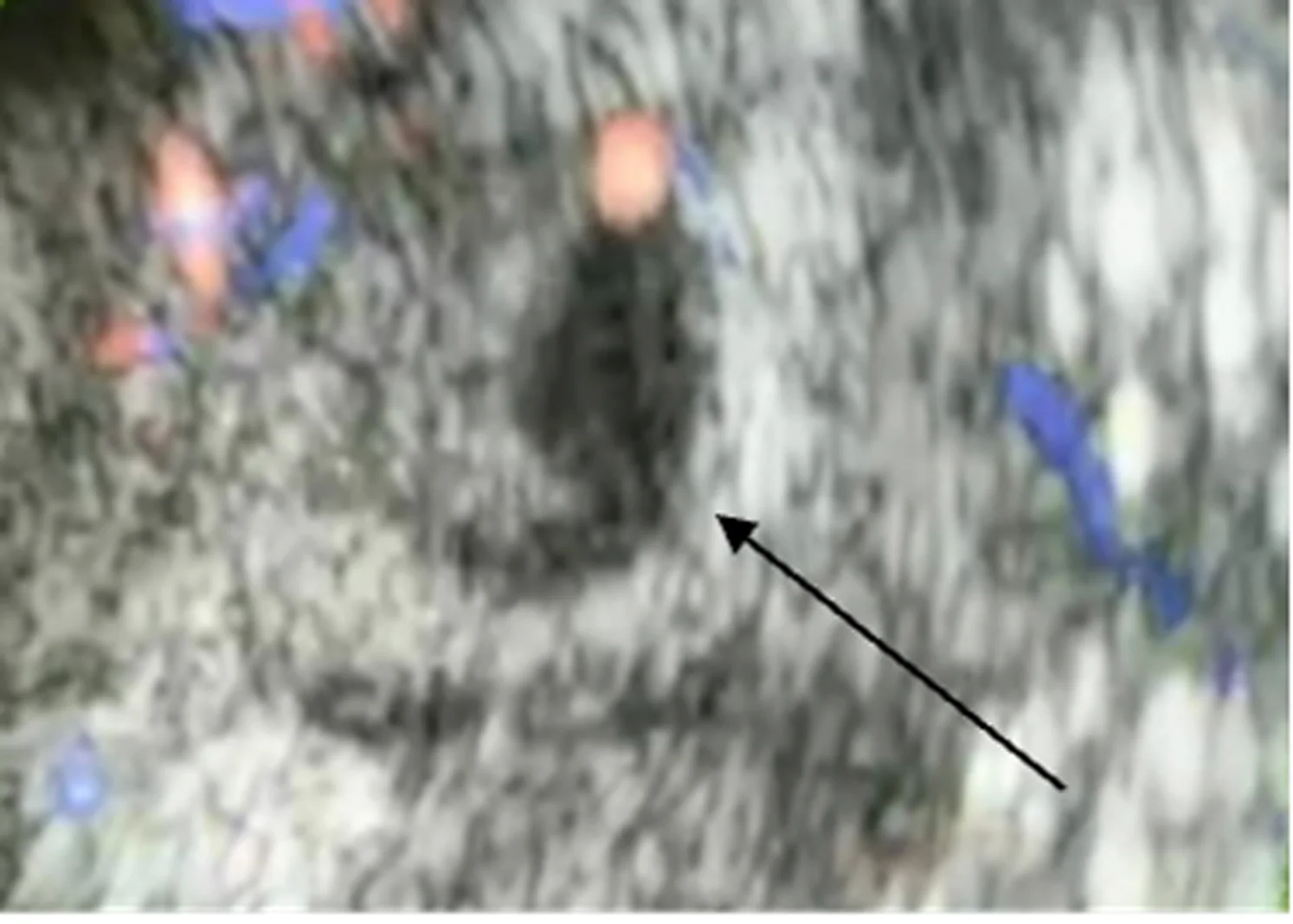
Endobronchial ultrasound showing vascular structure (black arrow) with turbulent, swirling flow concerning for a pseudoaneurysm.

*Answer:* Yes. In this case, color Doppler imaging during EBUS revealed turbulent, swirling flow adjacent to the area that was biopsied, exhibiting the classic yin-yang sign, an ultrasound finding consistent with bidirectional flow within a pseudoaneurysm. This characteristic Doppler pattern helped identify the vascular nature of the structure near the lesion and guided further management.

Color Doppler evaluation of the vascular structure revealed turbulent, swirling blood flow, consistent with a pseudoaneurysm (Video 1). A repeat CTA of the chest did not demonstrate active bleeding or acute vascular abnormalities. The patient was subsequently referred to interventional radiology, where angiography confirmed the presence of a right bronchial artery pseudoaneurysm (BAP) (Fig 3, Video 2). During the procedure, the BAP ruptured; however, coil embolization was successfully performed, achieving hemostasis. Cytology from the EBUS-guided biopsy was negative for malignancy, whereas fine needle aspirate cultures grew *Streptococcus anginosus* and *Prevotella baroniae*. The patient was treated for bacterial mediastinitis with an extended course of ampicillin-sulbactam. At 1-month follow up, there was complete resolution of the patient’s hemoptysis and CT scan of the chest demonstrated a reduction in the size of the mediastinal mass.

**Figure 3:**
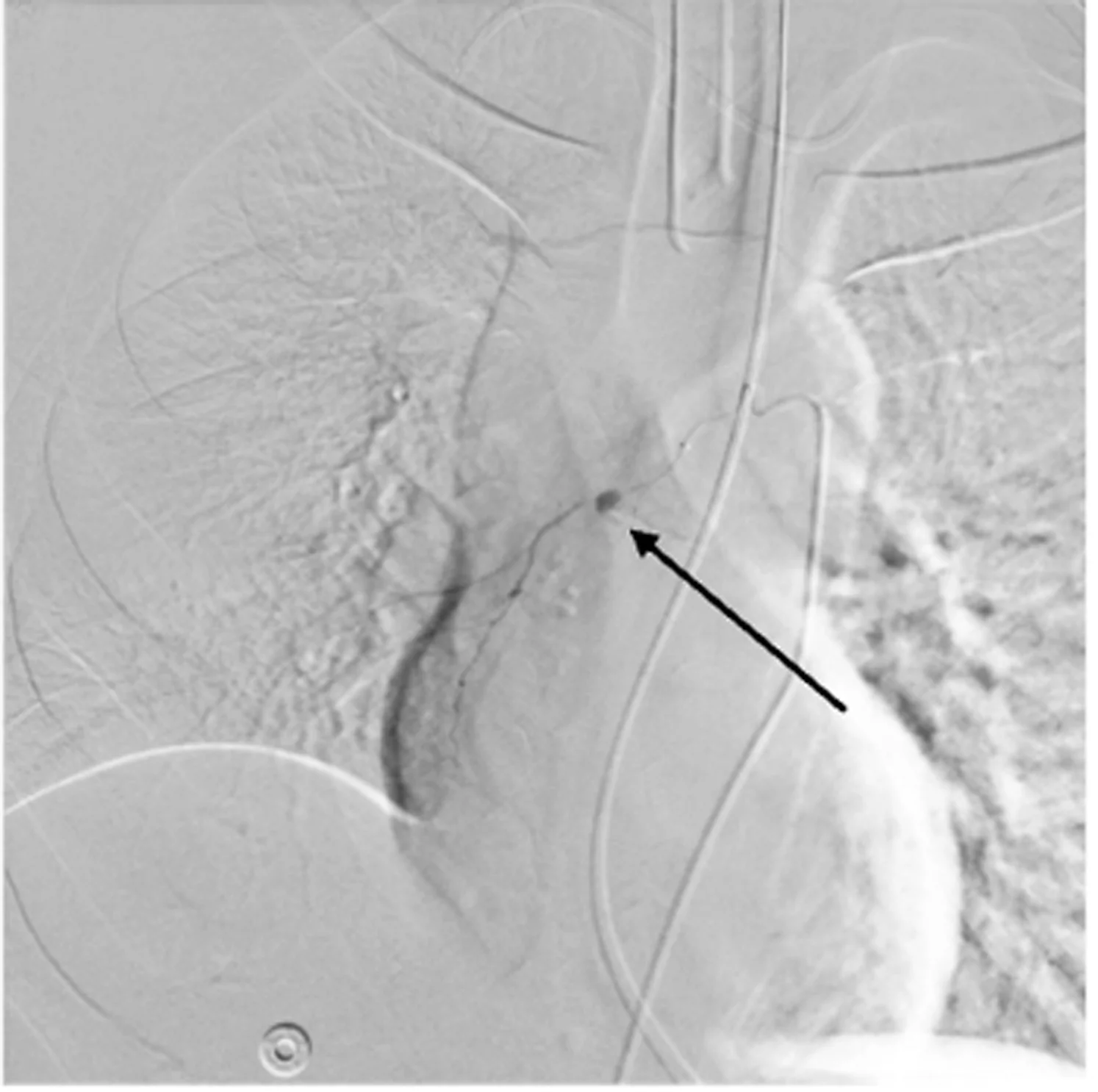
Interventional radiology angiography of the right bronchial artery pseudoaneurysm (black arrow).

## Discussion

In this case, bacterial mediastinitis led to compression and inflammation of adjacent bronchial and vascular structures, ultimately resulting in the formation of a right BAP. Mediastinitis can be classified as acute or chronic. Acute mediastinitis is most often infectious in origin and may arise from hematogenous or lymphatic spread, direct extension from surgical or procedural interventions, or invasion from adjacent structures.^1^ The causative organisms vary depending on the source, but bacterial pathogens are most common. In this patient, the likely source was an odontogenic or oropharyngeal infection, which frequently involves oral flora such as *Streptococcus* and *Bacteroides* species.

Chronic forms of mediastinitis include fibrosing mediastinitis and mediastinal granulomas.^2^ Fibrosing mediastinitis is a rare condition characterized by dense fibrosis within the mediastinum, often secondary to granulomatous or fungal infections such as histoplasmosis, aspergillosis, coccidioidomycosis, blastomycosis, or TB. Less common causes include silicosis, sarcoidosis, vasculitis, rheumatologic diseases, neoplasms (eg, Hodgkin lymphoma), and prior radiation therapy. In this case, granulomatous and fibrosing mediastinitis were considered, particularly in light of positive histoplasmosis IgG and aspergillus antigen. However, the absence of histoplasmosis IgM suggested these findings were due to prior exposure rather than active infection.

BAPs are rare, occurring in < 1% of selective bronchial angiograms.^3^ Common etiologies include infection, trauma, and malignancy, whereas less frequent causes include coagulopathies, connective tissue disorders, bronchiectasis, sarcoidosis, silicosis, iatrogenic injury, and vasculitis.^4^ Massive hemoptysis is often of bronchial arterial origin due to the higher systemic pressure and carries a high mortality risk.^5^ Compared with true aneurysms, pseudoaneurysms are more prone to rupture due to their thinner, less structured walls.^5^ In this case, the pseudoaneurysm ruptured during angiography but was successfully treated with coil embolization. Minimally invasive endovascular techniques such as this have largely replaced surgical approaches, offering improved outcomes and reduced mortality.^6^

Diagnosing BAPs can be challenging due to their small size and location. Prompt recognition is essential to prevent rupture and life-threatening hemorrhage. Bronchoscopists must be able to recognize vascular structures such as pseudoaneurysms and avoid biopsy in these lesions, as was done in this case. Although CTA is the first-line imaging modality, selective digital subtraction angiography remains the gold standard for detailed evaluation of bronchial vasculature.^3^^,^^6^ In this case, despite 2 CTAs, the BAP was not identified until EBUS was performed. Although EBUS is not traditionally used for vascular imaging, this case highlights its potential utility in identifying vascular abnormalities. Doppler ultrasound has been widely used in other clinical settings to detect bleeding complications and pseudoaneurysms.^7^ In symptomatic patients, duplex sonography has demonstrated 94% sensitivity and 97% specificity for detecting peripheral pseudoaneurysms after arterial puncture.^8^ Additionally, duplex Doppler has been successfully used to identify pseudoaneurysms during other endoscopic procedures.^9^

Pseudoaneurysms display characteristic sonographic features that vary by ultrasound modality. On B-mode imaging, turbulent or swirling flow may be observed, whereas color Doppler typically demonstrates the classic yin-yang sign of bidirectional flow. In the present case, this pattern was identified using a convex (curvilinear) array transducer integrated into the distal tip of the EBUS bronchoscope. Video 3 illustrates the activation of pulsed-wave Doppler during an EBUS examination, a technique that can also aid in identifying vascular structures throughout the procedure.

EBUS has become an essential tool in the evaluation of pulmonary, mediastinal, and hilar structures, allowing for real-time visualization during biopsy.^10^ Although color Doppler is routinely used during EBUS to identify bronchial arteries and avoid vascular injury, this case represents one of the first, to our knowledge, reported instances of a BAP identified using EBUS with color Doppler. This finding enabled targeted angiographic intervention and successful embolization. The patient recovered well, with resolution of hemoptysis and reduction in the size of the mediastinal mass.

## Reverberations

1. *Clinicians performing EBUS should be able to recognize the sonographic and Doppler features of pseudoaneurysms, including the classic yin-yang sign, pulsatility, and turbulent flow, to avoid inadvertent biopsy of vascular structures.*
2. *Routine use of color Doppler during EBUS improves procedural safety by helping to identify underlying vascular abnormalities, particularly in patients presenting with hemoptysis.*
3. *Early referral to interventional radiology for evaluation and management of suspected vascular lesions in the setting of massive hemoptysis can improve outcomes and reduce mortality.*

## Financial/Nonfinancial Disclosures

None declared.

## Supplementary Data

Video 1 Endobronchial ultrasound showing vascular structure with turbulent, swirling flow concerning for a pseudoaneurysm.

Video 2 Interventional radiology angiography of the right bronchial artery pseudoaneurysm.

Video 3 Instructional guide on how to perform pulsed wave Doppler during endobronchial ultrasound.
